# Grey matter volume alterations in CADASIL: a voxel-based morphometry study

**DOI:** 10.1007/s10194-012-0418-9

**Published:** 2012-02-03

**Authors:** Maria Camilla Rossi Espagnet, Andrea Romano, Filippo Carducci, Luigi Fausto Calabria, Martina Fiorillo, Francesco Orzi, Alessandro Bozzao

**Affiliations:** 1 Neurosciences and Sensory Organs (NESMOS) Department, Neuroradiology, Faculty of Medicine and Psychology, Sant’Andrea Hospital, University “Sapienza”, Via di Grottarossa 1035, 00139 Rome, Italy; 2 Research Institute (IRCSS) an Raffaele Pisana, Rome, Italy; 3 Laboratoy of Neuroimaging, Department of Physiology and Pharmacology, University “Sapienza”, Rome, Italy

**Keywords:** CADASIL, Dementia, DARTEL, VBM

## Abstract

CADASIL is a hereditary disease characterized by cerebral subcortical microangiopathy leading to early onset cerebral strokes and progressive severe cognitive impairment. Until now, only few studies have investigated the extent and localization of grey matter (GM) involvement. The purpose of our study was to evaluate GM volume alterations in CADASIL patients compared to healthy subjects. We also looked for correlations between global and regional white matter (WM) lesion load and GM volume alterations. 14 genetically proved CADASIL patients and 12 healthy subjects were enrolled in our study. Brain MRI (1.5 T) was acquired in all subjects. Optimized-voxel based morphometry method was applied for the comparison of brain volumes between CADASIL patients and controls. Global and lobar WM lesion loads were calculated for each patient and used as covariate-of-interest for regression analyses with SPM-8. Compared to controls, patients showed GM volume reductions in bilateral temporal lobes (*p* < 0.05; FDR-corrected). Regression analysis in the patient group revealed a correlation between total WM lesion load and temporal GM atrophy (*p* < 0.05; uncorrected), not between temporal lesion load and GM atrophy. Temporal GM volume reduction was demonstrated in CADASIL patients compared to controls; it was related to WM lesion load involving the whole brain but not to lobar and, specifically, temporal WM lesion load. Complex interactions between sub-cortical and cortical damage should be hypothesized.

## Introduction

CADASIL is an autosomal dominant hereditary disease characterized by a cerebral arteriopathy with subcortical infarcts and leucoencephalopathy. This monogenic small vessels disease (SVD) was first described in 1976, and is caused by dominant mutations in the NOTCH3 gene on chromosome 19 that encodes a 2,321-aminoacid-long transmembrane protein with 33 exons [1, 2].

The clinical picture may be heterogeneous, but usually the first manifestation of the disease is migraine with aura that occurs in 30% of patients, with a higher prevalence than in general population, followed by recurrent ischemic strokes, psychiatric disturbances and cognitive decline [3–5].

**Fig. 1:**
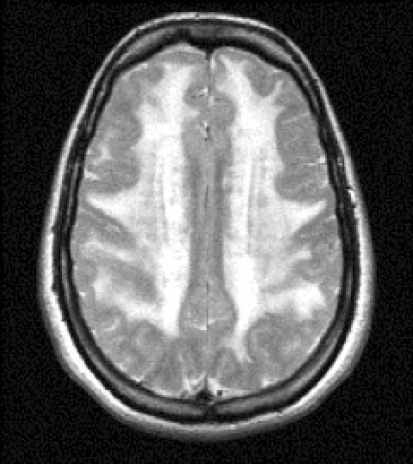
A typical pattern of distribution of white matter hyperintensities in a T2 MRI of a 42-year-old woman affected by CADASIL

White matter (WM) ischemic lesions represent an important imaging “marker” of the disease, usually appearing as subcortical and periventricular WM hyperintensities (WMHs) especially in the temporal lobe, external capsule, frontal and parietal lobe (Fig. 1) [6].

Previous studies have demonstrated that ischemic lesions correlate with cognitive performance and, thus, may represent a good imaging marker for the follow-up of disease progression [7]. Even if grey matter (GM) involvement has been described in CADASIL [8–11], so far there have been no studies investigating on GM volume differences between CADASIL patients and controls and on possible correlations between GM alterations and white matter lesions. Therefore, it is not clear whether the disease may cause structural changes in GM regions and if there are correlations between subcortical and cortical pathology.

Voxel based morphometry (VBM) is a whole brain and fully automated unbiased method that allows the detection of brain structural changes with a voxel by voxel analysis [12]. Recently, Diffeomorphic Anatomical Registration using Exponentiated Lie Algebra (DARTEL) has been introduced to achieve more accurate images’ registration and obtain a better evaluation of brain structural changes [13].

The purpose of our study was, therefore, to assess the presence of eventual GM volume changes in patients with CADASIL and to find correlations between WM lesions (lobar and whole brain) and GM conditions using VBM and DARTEL.

## Materials and methods

### Subjects

Subjects (both CADASIL patients and controls) were enrolled in our study from January 2010 until January 2011.

Fourteen subjects affected by CADASIL (nine females and five males with mean age 47 ± 8.3 years) were enrolled in our study. Inclusion criteria were the following:

1. Presence of a mutation on main exons of NOTCH3 gene involved in CADASIL disease (specifically exons 2, 3, 4, 5, 6, 11, 18, 19, 22 and 23) and/or skin biopsy positive for the presence of granular osmiophilic material (GOM);
2. Familiarity for CADASIL disease in 1st degree relatives;
3. Presence of diffuse WMHs on T2 sequences of magnetic resonance images [14].

None of the patients had focal neurological symptoms or deficits at the time of magnetic resonance imaging (MRI) examination.

Inclusion criteria for the control group were:

1. Absence of familiarity for vascular disorder;
2. No history of neurological disorder;
3. Normal neurological and general examination;
4. Mini Mental State Examination (MMSE) score >28;
5. No signal alterations on MR T1 and T2 sequences.

For control group 15 subjects (nine females and six males with mean age 47 ± 9 years) were initially enrolled but three of them were subsequently excluded because of movement artifacts on MR images.

The control group finally consisted of 12 healthy subjects (seven female and five male with mean age 42.3 ± 7.5 years).

MMSE was performed to assess cognitive status.

All subjects were right-handed.

Statistical differences between MMSE score of patient and control groups were evaluated by using a two-sample *t* test and standard deviation.

Local ethical committee approval and written informed consent were obtained from each participant.

### MRI acquisition

A single brain MRI scan of all subjects was done on a 1.5 T MR scanner (Magnetom, Siemens, Erlangen, Germany). The imaging protocol comprised 3D T1-weighted magnetization-prepared rapid acquisition gradient echo (MP-RAGE) sequence (TR 1,110 ms; TE 3.49 ms; matrix size 256 × 192; field of view 250 mm, section thickness 1 mm), T2 (TR 2,200 ms; TE 109 ms; matrix size 256 × 192; field of view 250 mm, section thickness 3 mm), and proton density (TR 2,200 ms; TE 24 ms; matrix size 256 × 192; field of view 250 mm, section thickness 3 mm).

All images were visually inspected by two neuroradiologists (AB and AR). Images with excessive motion artifact (i.e. indistinct sulci) were excluded. Three healthy subjects were excluded on this basis, leaving scans from 12 controls for analysis.

### Image processing

#### White matter lobar mask creation

In order to determine the volume of WM lesion load in CADASIL for frontal, parietal, temporal, limbic and occipital lobes, the corresponding 3D lobar WM masks were derived from the probabilistic brain atlas data provided by the Talairach Daemon software, developed by the Research Imaging Center of the University of Texas Health science Center San Antonio (UTHSCSA; http://www.talairach.org). These 3D masks were co-registered to the standard SPM T1 template.

#### White matter lesion load

In order to quantify WM lesion load in CADASIL patients, we used a fully automated algorithm for segmentation of lesions from T1, T2 and PD images. The method performs intensity-based tissue classification using a stochastic model for normal brain images and simultaneously detects brain lesions (on PD and T2) as outliers that are not well explained by the model [15]. Although the algorithm is fully automatic, an appropriate Mahalanobis distance threshold had to be chosen in advance, to appropriately classify these outliers. In this study, we used a Mahalanobis threshold of 4.1 because we considered it to be the best threshold to detect WMHs without involving other structures. Finally, a 3D WM lesion map was created for each patient. These lesion maps were co-registered to the same standard SPM T1 template used in the WM lobar mask creation step.

The 3D lobar masks were applied on WM lesion maps, to produce binary WMH lobar maps for each patient. The quantification of global and lobar WM lesion loads was calculated using the ImageJ tool (http://rsweb.nih.gov/ij). In order to have an individual WM lobar lesion load index, lobar WM lesion load values of each patient were normalized with respect to the volume of the corresponding lobar mask. Lobar and total lesion load indexes were then used as covariates in subsequent regression analysis.

### Voxel based morphometry

GM aberrations in CADASIL were analyzed on T1 images by means of Voxel-based morphometry (VBM) that was performed using the Statistical Parametric Mapping 8 package (SPM8; Wellcome Trust Center for Neuroimaging, Oxford, England; http://www.fil.ion.ucl.ac.uk/spm) running under MATLAB R2011a (The Mathworks, Sherborn, MA, USA) and the diffeomorphic anatomical registration using exponentiated Lie algebra (DARTEL; Friston 2007), method used to achieve a more precise images’elaboration [13].

Before VBM analysis, the WM global lesion load mask of each patient was removed from co-registered T1 images to avoid misclassification errors in the VBM segmentation step [17]. T1 images were then segmented in GM, WM, and cerebrospinal fluid (CSF) partitions. The segmented images were visually inspected by a team of two experts and, then, imported to DARTEL for warping procedure, and finally iteratively aligned to the average template. During DARTEL warping, the segmented images were modulated with Jacobian determinates to preserve volume changes. Normalized modulated GM was finally smoothed with an 8-mm full width half maximum Gaussian kernel. Expert users carefully evaluated each step to check for normalization or segmentation errors.

### Statistics

In order to evaluate any difference in MMSE and total intracranial volume (TIV) between CADASIL patients and controls, mean values (MVs), standard deviations (SDs) and Student’s *t* tests were calculated (statistical threshold set to *p* < 0.05) using SAS Software Package version 9.2 (SAS Institute Inc., NC, USA)

Differences between patients and controls for VBM analysis were evaluated using a general linear model (GLM), through pre-processed images. Two-sample *t* test was applied to provide voxel-wise group comparisons of volumes of GM co-varying for age, gender and TIV to account for possible confounding factors. TIV was calculated as the sum of GM, WM and CSF volumes, provided by VBM. False discovery rate (FDR) approach was chosen to minimize the risk of making type I errors, and the threshold was set to *p* < 0.05.

To evaluate any correlations between global or lobar WM lesion load and GM structural changes in the patient group, we used a multiple regression analysis integrated in SPM basic models. Age, gender and TIV were used as a covariate of no interest whilst WM lesion load as a covariate of interest, to highlight regions with proportionally less GM volume in patients with more WM lesion load.

Using the tool FSL-view of FSL software and Talairach Demon Labels (http://www.fmrib.ox.ac.uk/fsl), the anatomic location of significant clusters was detected.

## Results

Two patients had a MMSE score under 26. The average and SD of MMSE score were 28 ± 2 in the patient group and 29.6 ± 1.3 in the controls. There were no statistically significant differences in MMSE scores between patients and controls (*p* = 0.21).

There were no significant differences (*p* = 7.45) in TIV between CADASIL patients (MV and SD 1,850 ± 54.3) and controls (MV and SD 1,930 ± 42.6).

**Fig. 2:**
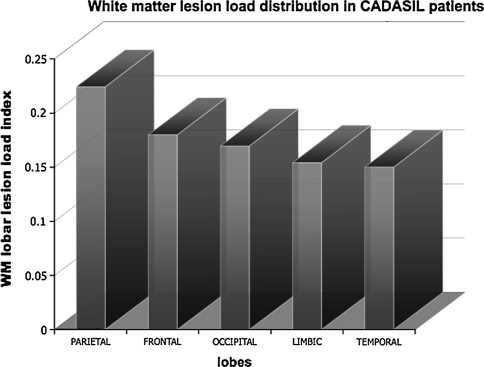
The graphic shows white matter hyperintensities distribution in CADASIL patients within cerebral lobes. Lesion load is expressed as the ratio between each subject lobar lesions volume and the volume of the lobar mask (*y* axis). On *x* axis the mean value of lobar (*right* and* left*) indexes is reported

WMHs in CADASIL patients were more evident in the parietal lobes, followed by frontal, occipital, limbic and temporal regions (Fig. 2).

**Fig. 3:**
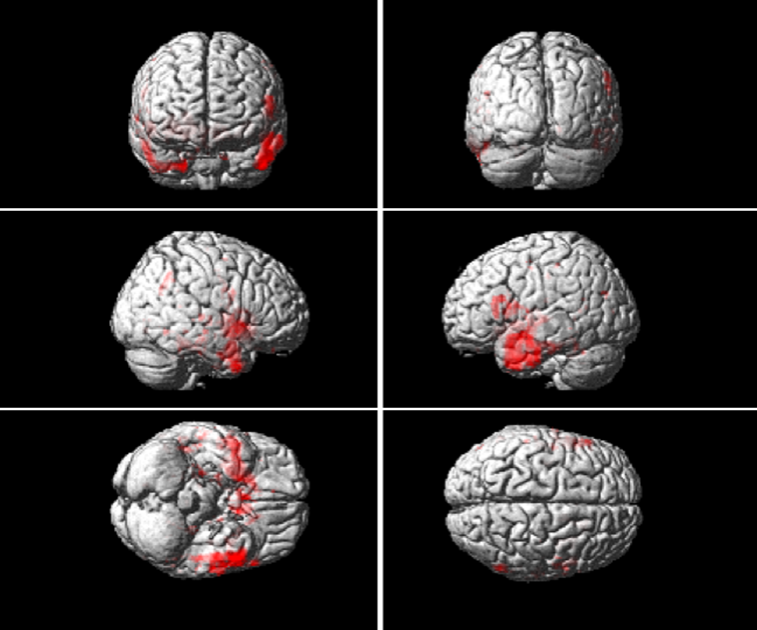
Regions of* grey matter* volume reduction (*red*) in CADASIL versus healthy controls (color figure online)

VBM analysis revealed a decreased GM volume in the patients group compared to controls, in the temporal lobes (middle temporal gyrus), bilaterally and in caudate nucleus, *p* < 0.05 FDR-corrected (Table 1; Fig. 3).

**Table 1:** Regions of decreased grey matter volume in CADASIL compared with control

Cluster	MNI-space			*K* value	*p* value	*Z* value	Side	Lobe	Anatomic region
	*x*	*y*	*z*						
1	−51	3	−36	1,193	0.000*	3.95	L	Temporal	Middle temporal gyrus
2	51	6	−39	427	0.02*	3.66	R	Temporal	Middle temporal gyrus
3	−57	22	6	150	0.27	4.3	L	Frontal	Inferior frontal gyrus
4	18	−27	2	50	1	4.15	R		Thalamus (Pulvinar)
5	65	−26	2	30	1	4.05	R	Temporal	Superior temporal gyrus
7	30	9	−11	356	0.000*	5.21	R		Caudate nucleus

*MNI* Montreal Neurological Institute, *x*,* y*,* z* coordinates of the primary maximum of the cluster, *K* *value* number of voxels in the cluster, *L* left hemisphere, *R* right hemisphere

* Clusters surviving a statistical threshold of *p* < 0.05, FDR corrected

**Fig. 4:**
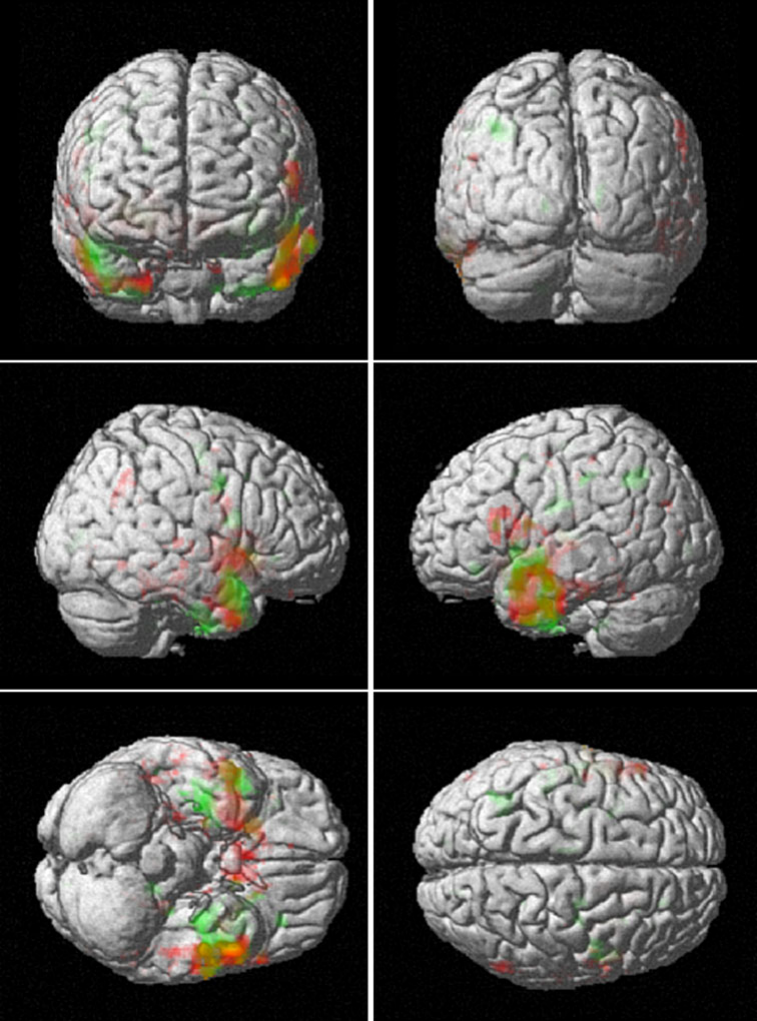
Regions of decreased* grey matter* (GM) volume from the comparison of CADASIL patients with healthy controls (in *red*) and regions of GM volume reduction related to total white matter (WM) lesions load of each patient (*green*). The concordance of GM volume reduction in the two analyses underlines the relevance of total WM lesion load in its development (color figure online)

The regression analysis performed only in the patient group revealed a correlation between total WM lesion load (used as independent variable) and reduction in temporal lobes grey matter volume (the dependent variable) (Table 2; Fig. 4).

**Table 2:** Regions of decreased grey matter volume in CADASIL patients correlated to white matter lesions load

Cluster	MNI-space			*K* value	*p* value	*Z* value	Side	Lobe	Anatomic region
1	−63	−6	−21	1,450	0.001*	3.84	L	Temporal	Inferior temporal gyrus
2	18	14	−11	100	0.09	2.9	R		Putamen
3	−32	55	−12	50	0.5	2.8	L	Frontal	Middle frontal Gyrus
4	50	8	−24	907	0.012*	3.24	R	Temporal	Superior temporal gyrus
5	5	−78	8	63	0.6	3.19	R	Occipital	Cuneus

*MNI* Montreal Neurological Institute, *x*, *y*, *z* coordinates of the primary maximum of the cluster, *K* *value* number of voxels in the cluster, *L* left hemisphere, *R* right hemisphere

* Clusters surviving a statistical threshold of *p* < 0.05, uncorrected

When lobar WM lesion load (for each lobe) was set as independent variable, no regions of significant alterations in GM volumes were observed in the regression analysis.

## Discussion

Subcortical WM ischemic injury is the main pathological sign of CADASIL, a model of “pure” monogenic small-vessel disease. Currently, conventional MRI plays a key role in the evaluation of CADASIL disease, showing subcortical WMHs on T2-weighted images, LL and cerebral microhemorrages (CMs), visible, respectively in T1 and gradient echo sequences [1, 16–18]. WMHs are one of the first imaging signs of CADASIL often appearing before clinical onset and localized in the WM of parietal and frontal lobes, external capsules and especially in temporal regions with a suggested 90% sensitivity and 100% specificity [6, 19–21]. Although the temporal location is considered typical for the disease, it was not the prevalent one in our case series (Fig. 2).

Different studies have investigated the pathological significance of WM lesions and the pattern of their distribution, to speculate on possible pathogenic hypotheses and indicate a specific imaging marker of the disease. Other studies have been addressed to find correlations between these radiological findings and the clinical status, examined by neuropsychological tests and specific scoring systems for disability [6, 22]. Viswanathan et al. [22] have used an integrated multi-modal model to determine the impact of WMHs, lacunar lesions, microhemorrages, mean apparent diffusion coefficient (ADC) and brain parenchymal fraction (BPF) on cognition and disability. The main finding of their study was the strong correlation between brain atrophy and cognitive impairment in CADASIL represented by a statistical association between global cognitive function (assessed with MMSE) and BPF.

Recently, brain atrophy has emerged as an important characteristic of several microangiopathies, being a biomarker of the cognitive status in CADASIL [7, 20, 22, 23]. In a longitudinal analysis, Peters et al. [23] demonstrated that the annualized rate of brain volume loss is almost two to three times higher in CADASIL patients than in healthy subjects of similar age, and that the annual percent change of brain volume is statistically related to global cognitive performance and disability. Liem et al. [24] have obtained different results; these authors demonstrated an equal rate of brain volume loss in CADASIL patients compared to controls. Thus, the real importance of brain atrophy in the pathogenesis and clinical progression of CADASIL remains controversial. Recently, cortical damage was shown to occur from neuronal apoptosis in a postmortem study by Viswanathan et al. [25].

Different volumetric approaches, mainly based on a priori hypotheses, have been used to assess the involvement of specific cortical regions in the disease and their relation to patients’ clinical status [8, 10, 11]. All these studies confirmed the clinical relevance of GM involvement, underlying the inhomogeneity of cortical alterations. In our study, we looked for GM volume changes in patients with CADASIL by comparing them to healthy controls. We used VBM, a fully automated technique, that provides a whole brain and observer-independent analysis and allows the identification of differences in local composition of brain tissue in vivo and voxel-by-voxel in a non-invasive way [12]. This method has recently improved with the introduction of DARTEL analysis that allows the identification of structural changes with a more accurate inter-subject alignment [13]. Our analysis demonstrated bilateral GM atrophy in the temporal lobes of patients with CADASIL (Fig. 3; Table 1) confirming GM involvement in the disease. Temporal lobe involvement was described in a previous VBM analysis but only on WM. In that study, Auer et al. [26] used a statistical parametric mapping analysis to investigate WM alterations in CADASIL patients, comparing them to subjects affected by subcortical arteriosclerotic encephalopathy and showing a specific involvement of temporal regions in the first ones. Yamamoto et al. [9] confirmed, in a post-mortem analysis, temporal lobe damage; WMHs correlated with WM rarefaction, myelin depletion and periventricular spaces enlargement. Cortical changes affecting temporal lobes were shown by Jouvent et al. These authors demonstrated a reduction of depth and surface of superior temporal, superior frontal, cingulate and central sulci, which appeared to be correlated with cognitive and disability scales [10].

The investigation of eventual relationships between cortical changes and WM lesion load in CADASIL was the other target of our study. Regression analyses were performed in the patient group using total WM lesion load and lobar (temporal, parietal, occipital, frontal and limbic) lesion load as independent variables for each regression analysis performed in SPM.

We demonstrated a correlation between total WMHs and decreased cortical temporal volume (Fig. 3; Table 2). On the other hand, when lobar lesion load (for example temporal WMHs) was set as covariate of interest, no statistically significant correlations with GM volume alterations appeared. These results suggest that no correlation exists between WM and GM damage within the same cerebral lobe and specifically in the temporal lobe.

The relationship between WMHs and brain atrophy in CADASIL is not fully understood. Jouvent et al. [16] showed a lack of association between the extent of WMHs and global brain atrophy demonstrating, on the other hand, a relationship between LL and brain parenchymal fraction (BPF), as estimate of cortical atrophy. More recently, Viswanathan et al. [25] have shown that the presence of cortical neuronal apoptosis, in cerebral pathological samples of CADASIL subjects, was related to different types of subcortical WM damage (i.e. WMHs, LL and CMs) and especially to axonal damage. These results suggest that in CADASIL other microstructural alterations, such as LL, demyelination, axonal loss and CMs may contribute, beyond WMHs, to GM damage through deafferentation and retrograde neuronal degeneration. Considering this evidence and our results altogether, we suggest that the extent of WMHs and cortical temporal lobes atrophy progresses simultaneously during the course of the disease. They could be the result of vascular impairment and microstructural disorganization, both leading to cortical neuronal apoptosis through deafferentiation.

Our study has some limitations. The first is the population size: too small to obtain higher statistic significance, especially for the regression analysis. Other important limitations are that the study is cross-sectional and consequently the nature of the relationship found between WM lesion load and GM temporal atrophy cannot be inferred. Furthermore, we only investigated the relation between GM atrophy and lobar or global WMHs load, without considering LL and CMs.

In addition, neuropsychological evaluation and disability assessment were not obtained (only MMSE was tested and did not differ from control group), thus we did not determine the clinical relevance of temporal cortical atrophy in our population.

In conclusion, the results of our study demonstrated that, using VBM with DARTEL, patients affected by CADASIL show a selective decrease in GM temporal volume. This is related to the extent of WMHs in the whole brain, but not to regional lesion load. This suggests that vascular impairment and microstructural disorganization together may lead to cortical neuronal apoptosis through deafferentation.

## References

[1] Chabriat H, Joutel A, Dichgans M, Tournier-Lasserve E, Bousser MG (2009). Cadasil. Lancet Neurol.

[2] Joutel A (2010). Pathogenesis of cadasil: transgenic and knock-out mice to probe function and dysfunction of the mutated gene, notch3, in the cerebrovasculature. Bioessays.

[3] Liem MK, Oberstein SA, Van der Grond L (2010). Cadasil and migraine: a narrative review. Cephalalgia.

[4] Di Piero V, Bonaffini N, Altieri M (2004). Migraine and cerebrovascular disease. J Headache Pain.

[5] Sacco S, Rasura M, Cao M, Bozzao A, Carolei A (2009). CADASIL presenting as status migrainosus and persisting aura without infarction. J Headache Pain.

[6] Singhal S, Rich P, Markus HS (2005). The spatial distribution of MR imaging abnormalities in cerebral autosomal dominant arteriopathy with subcortical infarcts and leukoencephalopathy and their relationship to age and clinical features. Am J Neuroradiol.

[7] Viswanathan A, Gschwendtner A, Guichard JP, Buffon F, Cumurciuc R, O’Sullivan M, Holtmannspotter M, Pachai C, Bousser MG, Dichgans M, Chabriat H (2007). Lacunar lesions are independently associated with disability and cognitive impairment in cadasil. Neurology.

[8] O’Sullivan M, Ngo E, Viswanathan A, Jouvent E, Gschwendtner A, Saemann PG, Duering M, Pachai C, Bousser MG, Chabriat H, Dichgans M (2009). Hippocampal volume is an independent predictor of cognitive performance in cadasil. Neurobiol Aging.

[9] Yamamoto Y, Ihara M, Tham C, Low RW, Slade JY, Moss T, Oakley AE, Polvikoski T, Kalaria RN (2009). Neuropathological correlates of temporal pole white matter hyperintensities in cadasil. Stroke.

[10] Jouvent E, Mangin JF, Porcher R, Viswanathan A, O’Sullivan M, Guichard JP, Dichgans M, Bousser MG, Chabriat H (2008). Cortical changes in cerebral small vessel diseases: a 3D MRI study of cortical morphology in cadasil. Brain.

[11] Stromillo ML, Dotti MT, Battaglino M, Mortella M, Bianchi S, Plewnia K, Pantoni L, Inzitari D, Federico A, De Stefano N (2009). Structural and metabolic brain abnormalities in preclinical cerebral autosomal dominant arteriopathy with subcortical infarcts and leucoencephalopathy. J Neurol Neurosurg Psychiatry.

[12] Ashburner J, Friston KJ (2000). Voxel-based morphometry—the methods. Neuroimage.

[13] Ashburner J (2007). A fast diffeomorphic image registration algorithm. Neuroimage.

[14] Ampuero I, Alegre-Abarrategui J, Rodal I (2009). On the diagnosis of CADASIL. J Alzheimer Dis.

[15] Van Leemput K (2001). Automated segmentation of multiple sclerosis lesions by model outlier detection. IEEE Trans Med Imaging.

[16] Chabriat H, Joutel A, Dichgans M, Tournier-Lasserve E, Bousser MG (2009). Cadasil. Lancet Neurol.

[17] Rocca MA, Ceccarelli A, Falini A, Colombo B, Tortorella P, Bernasconi L, Comi G, Scotti G, Filippi M (2006). Brain gray matter changes in migraine patients with T2-visible lesions: a 3-T MRI study. Stroke.

[18] Jouvent E, Viswanathan A, Mangin JF, O’Sullivan M, Guichard JP, Gschwendtner A, Cumurciuc R, Buffon F, Peters N, Pachai C, Bousser MG, Dichgans M, Chabriat H (2007). Brain atrophy is related to lacunar lesions and tissue microstructural changes in cadasil. Stroke.

[19] O’Sullivan M, Jarosz JM, Martin RJ, Deasy N, Powell JF, Markus HS (2001). MRI hyperintensities of the temporal lobe and external capsule in patients with CADASIL. Neurology.

[20] Kalimo H, Ruchoux M-M, Viitanen M, Kalaria RN (2002). CADASIL: a common form of hereditary arteriopathy causing brain infarcts. Brain Pathol.

[21] Markus HS, Martin RJ, Simpson MS, Dong YB, Ali N, Crosby AH, Powell JF (2002). Diagnostic strategies in CADASIL. Neurology.

[22] Viswanathan A, Godin O, Jouvent E, O’Sullivan M, Gschwendtner A, Peters N, Duering M, Guichard JP, Holtmannspotter M, Dufouil C, Pachai C, Bousser MG, Dichgans M, Chabriat H (2008). Impact of MRI markers in subcortical vascular dementia: a multi-modal analysis in cadasil. Neurobiol Aging.

[23] Peters N, Holtmannspotter M, Opherk C, Gschwendtner A, Herzog J, Samann P, Dichgans M (2006). Brain volume changes in cadasil: a serial MRI study in pure subcortical ischemic vascular disease. Neurology.

[24] Liem MK, Lesnik Oberstein SA, Haan J, van der Neut IL, van den Boom R, Ferrari MD, van Buchem MA, van der Grond J (2008). Cerebral autosomal dominant arteriopathy with subcortical infarcts and leukoencephalopathy: progression of MR abnormalities in prospective 7-year follow-up study. Radiology.

[25] Viswanathan A, Gray F, Bousser MG, Baudrimont M, Chabriat H (2006). Cortical neuronal apoptosis in cadasil. Stroke.

[26] Auer DP, Putz B, Gossl C, Elbel G, Gasser T, Dichgans M (2001). Differential lesion patterns in CADASIL and sporadic subcortical arteriosclerotic encephalopathy: MR imaging study with statistical parametric group comparison. Radiology.

